# Supraglottic stenosis as a late complication of radiotherapy: a case report

**DOI:** 10.31744/einstein_journal/2022RC0035

**Published:** 2022-08-24

**Authors:** Claudiney Cândido Costa, Sarah Vidal da Silva, Mateus Capuzzo Gonçalves, Hugo Valter Lisboa Ramos

**Affiliations:** 1 Universidade Federal de Goiás Goiânia GO Brazil Universidade Federal de Goiás, Goiânia, GO, Brazil.

**Keywords:** Laryngostenosis, Radiotherapy, Nasopharyngeal carcinoma

## Abstract

Nasopharyngeal carcinoma is rare and affect mainly men between the fourth and sixth decades of life. The clinic is characterized to be nonspecific and the main complaints or findings related to this disease are: cervical mass, aural dysfunction, and headache. The basis of treatment is radiotherapy that involves a wide field of irradiation of normal tissues, which usually generates sequelae with direct implications for quality of life. We report a case of a nasopharyngeal carcinoma treated with radiotherapy and chemotherapy that evolved, after 8 years, into supraglottic stenosis. We emphasize the relevance of clinical follow-up after radiotherapy, particularly due to the late sequelae and the relevance of using radiotherapy devices with a more focal cancer field, in order to minimize complications.

## INTRODUCTION

Nasopharyngeal carcinoma is rare and presents an incidence of 1:100,000. This disease has a higher prevalence in Asians, affects 3 men to every woman, and shows a peak incidence between the fourth and sixth decades of life. The clinic is characterized by non-specificity so that, the main complaints or findings related to the disease are: neck mass (76%), aural dysfunction (62%), and headache (35%). Diagnosis occurs with locally advanced disease in 75-90% of cases, and commonly along with lymph node metastases.^(1)^ The mainstay of treatment is radiotherapy that must be added to chemotherapy from stage 2 onwards. Radiotherapy in nasopharyngeal carcinoma involves a wide field of irradiation of normal tissues and this is associated with doses that exceed the tolerance for radiation of these tissues. There are high rates of toxicity in up to ninety days (early) or beyond this period (late), which are potentiated with the use of chemotherapy, and promote a significant decrease in quality of life.^(2)^ The most common late sequelae resulting from radiotherapy in rhinopharyngeal carcinoma involve temporal lobe, optic nerve, trismus, osteoradionecrosis, and they often show a mean time of presentation of 18 months.^(3)^

## CASE REPORT

**Figure 1:**
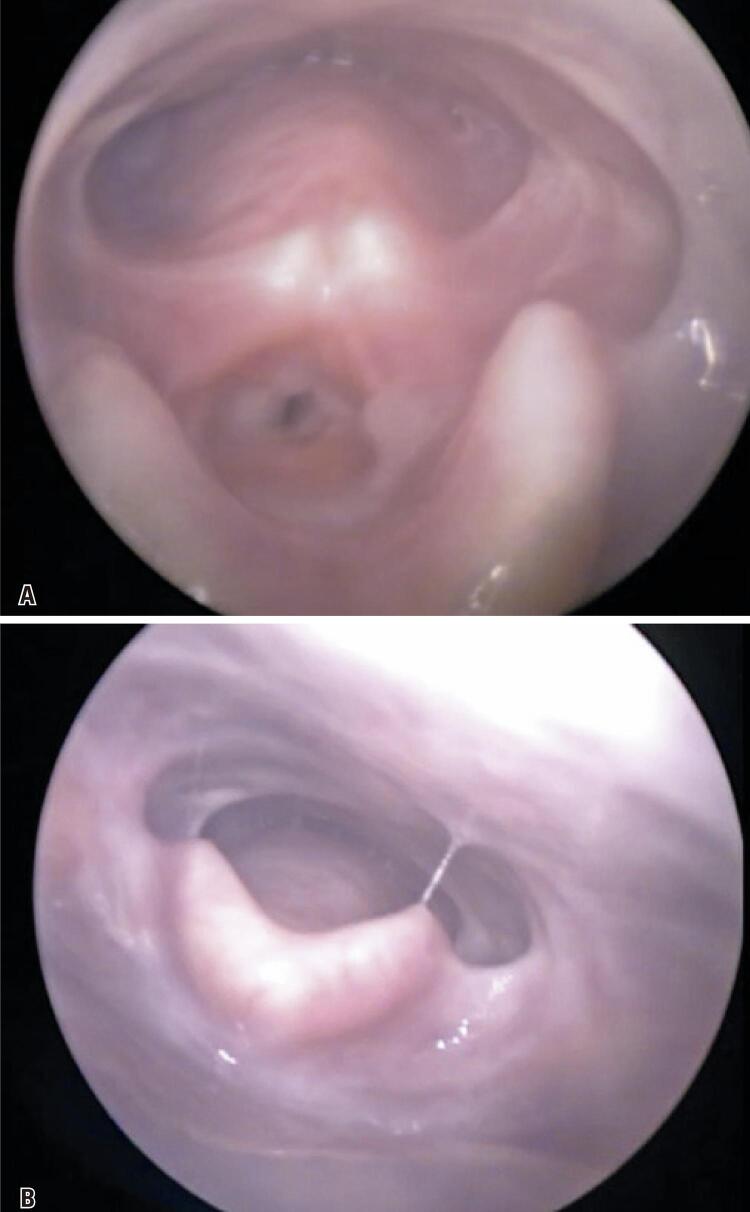
Video laryngoscopy with 70° optics. (A) Circular supraglottic stenosis at the level of the vestibular folds; (B) Signs of diffuse fibrosis involving epiglottic cartilage and vallecula

**Figure 2:**
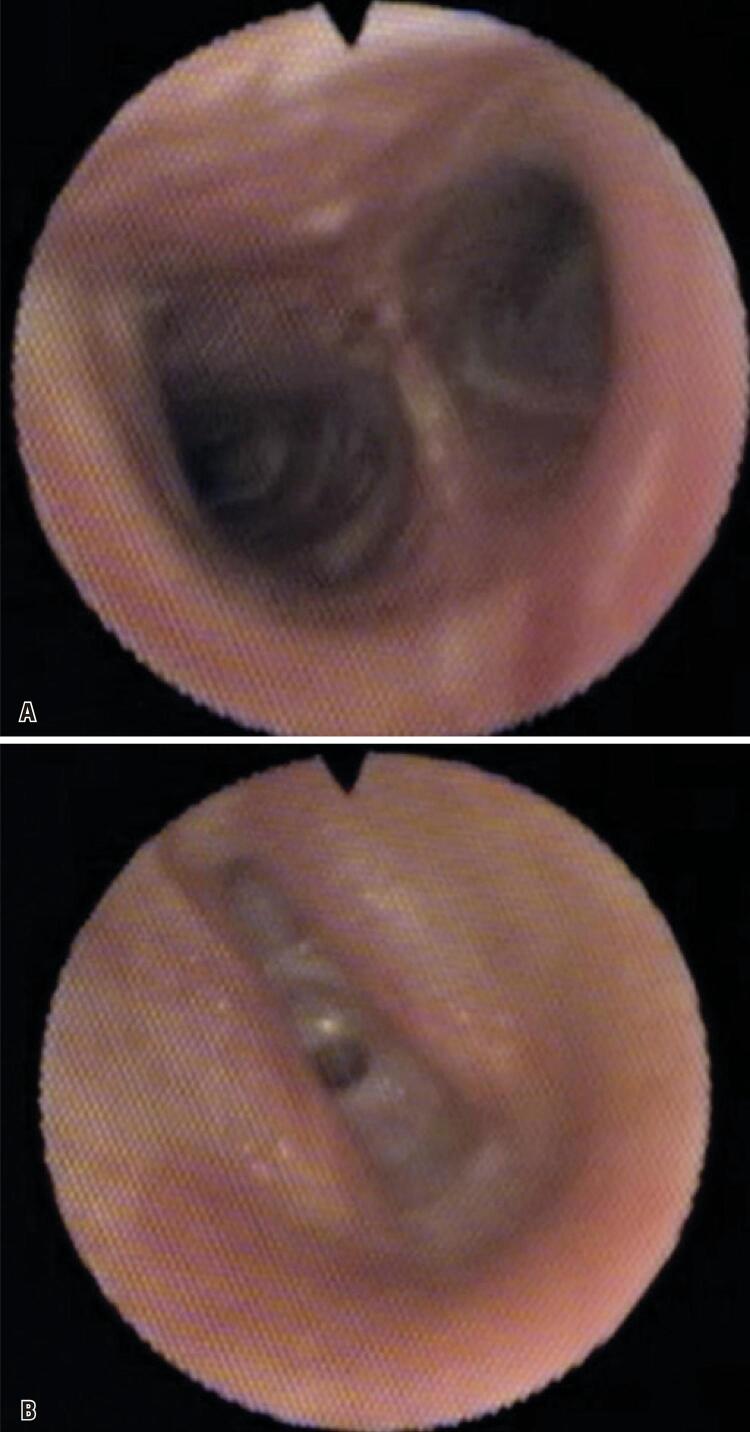
Videotracheostomy with flexible fiber (through the tracheostoma): Late supraglottic stenosis 8 years after radiotherapy of the rhinopharynx and neck to treat undifferentiated carcinoma of the rhinopharynx. (A) Visualization of the carina and the right and left main bronchi, without alterations; (B) Videotracheostomy showing the vocal folds and the circular supraglottic stenosis

This was a 39-years-old woman that in February 2006 started to present nasal obstruction on the right side associated with postnasal drip, which evolved after a month with aural fullness, hearing loss on the right, and autophonia. Nasofibrolaryngoscopy showed a tumor mass in the rhinopharynx with occlusion of the ostium of the right auditory tube. An incisional biopsy was performed and the result was nasopharyngeal carcinoma in situ (T1N0M0). In June 2006, she underwent 35 sessions of 2D radiotherapy with 70Gy in primary and 30Gy in lymphatic drainage associated with 3 sessions of chemotherapy. In December 2006, the patient reported odynophagia and dysphagia. During the nasofibrolaryngoscopy exam, diffuse edema involving the epiglottis, aryepiglottic folds, arytenoid cartilages, and vestibular folds were observed. A complete regression of the condition was seen after treatment using amoxicillin with clavulanate, omeprazole, and speech therapy. In 2013, she reported dyspnea at rest, cough, and dysphagia. A videolaryngoscopy was performed with a 70° optic. This showed circular supraglottic stenosis of approximately 95% of the lumen at the level of the vestibular folds, hypoplasia of the epiglottis, and alteration of the anatomy of the valleculae (Figure 1A and B). No changes were observed in the upper digestive endoscopy, requiring tracheostomy to ensure airway patency. A videotracheostomy performed one month after tracheostomy revealed normal carina and bronchi sources and supraglottic ring stenosis (Figure 2A and B). Currently, the patient awaits supraglottoplasty and continues with tracheostomy and phonatory valve and presents good adaptation.

## DISCUSSION

Radiotherapy represents the first line in the treatment of nasopharynx carcinoma, but it is related to several early adverse effects such as xerostomia, cutaneous fibrosis. Late complications include advanced age of the patient, clinical staging and chemotherapy as risk factors. They tend to appear in the first 5 years of clinical follow-up and they are related to the dose used in healthy tissue and its respective tolerability.^(2)^ The patient of this case report was treated with an association between radiotherapy and chemotherapy, an approach that may have contributed to the late presentation of supraglottic stenosis.

Stevens et al., in a series of 8 cases with supraglottic stenosis observed that the most common cause was external beam irradiation followed by autoimmune diseases. In addition, they reported that time of presentation after radiotherapy was 18-120 months, which is a similar finding to our clinical case that presentation of supraglottic stenosis occurred late to treatment, *i.e.*, after 96 months.^(4)^

Supraglottic stenosis after radiotherapy is rare, corresponding to 3% of laryngotracheal stenosis, the clinical picture involves dyspnea, dysphagia, stridor, and changes in voice resonance. Depending on the degree of stenosis, orotracheal intubation can be a challenge, as can tracheostomy in a pre-irradiated area.^(5)^ Our patient presented significant progressive ventilatory impairment associated with inspiratory and expiratory stridor, even at rest, which required intervention to ensure ventilation three tracheostomy. This procedures was performed in a surgical environment with local anesthesia, since it was not possible to perform the orotracheal intubation, and had no intercurrences.

Tissue-modulated intensity radiotherapy discriminates the radiation dose delivered to the peritumoral area and to the tumor, promotes better preservation of normal tissues and, consequently, reduces the incidence of complications of this treatment.^(6,7)^

In 2006, the patient was submitted to a 2D radiotherapy modality, which dose inflicted on healthy tissues surrounding the tumor was higher due to the unavailability of the tissue-modulated intensity radiotherapy system. We believe that this may have been a factor that contributed to the unfavorable outcome herein presented.

The treatment of post-radiotherapy stenosis involves several options such as: balloon dilation, open surgical or videolaryngoscopic approach with carbon dioxide (CO_2_) or potassium-titanyl-phosphate laser, and application of mitomycin C or corticosteroids. The control of laryngopharyngeal reflux is essential, since its presence can contribute to the persistence or development of supraglottic stenosis. In most severe cases of upper airway stenosis, as reported here, tracheostomy is usually necessary to ensure airway patency.^(4,7)^ In this case report, an initial approach by videolaryngoscopy was proposed with the use of CO_2_ laser, aiming to decannulate the patient, however, the patient still unsure about the benefits of the procedure.

## CONCLUSION

Late complications related to radiotherapy are rare, whether or not associated with chemotherapy. Patients with tumors in the head and neck region, submitted to the treatment, are subject to the tumor appearance. When possible, tissue-modulated intensity radiotherapy should be indicated due to its lower morbidity and mortality. The follow-up of patients should be longer in an attempt to carry out an early diagnosis and treatment.
